# A modified physiologic test for bronchopulmonary dysplasia: a clinical tool for weaning from CPAP and/or oxygen-therapy the premature babies?

**DOI:** 10.1186/s13052-018-0582-x

**Published:** 2019-01-04

**Authors:** Giovanni Vento, Valentina Vendettuoli, Claudia Aurilia, Milena Tana, Chiara Tirone, Alessandra Lio, Piero Giuseppe Matassa, Francesca Priolo, Piero Catenazzi, Mirta Corsello, Enrico Zecca, Fabio Mosca

**Affiliations:** 1 Division of Neonatology, Department of Pediatrics, Fondazione Policlinico Universitario A. Gemelli IRCCS, Rome, Italy; 2 Università Cattolica del Sacro Cuore, Largo A. Gemelli, 8, 00168 Rome, Italy; 3 Neonatal Intensive Care Unit, Department of Clinical Sciences and Community Health, Fondazione IRCCS Ca’ Granda Ospedale Maggiore Policlinico, Università degli Studi di Milano, Milan, Italy

**Keywords:** Physiologic test, Preterm infants, Respiratory support weaning

## Abstract

**Background:**

A physiologic test for estimating BPD rate has been developed by Walsh and collaborators. Actually there are not standard criteria for weaning from CPAP and/or oxygen therapy the premature babies. Aim of this study was to verify if a physiologic test, modified respect to that developed by Walsh and collaborators for estimating BPD rate, can be used as a clinical tool for weaning the premature babies from CPAP and/or oxygen therapy.

**Methods:**

Neonates with BW 500–1250 g and GA ≤ 32 weeks, receiving FiO_2_ ≤ 0.30 by hood or CPAP, were prospectively studied at 28 days of life and at 36 weeks of postmestrual age. The test was performed in 3 steps: baseline, challenge (FiO_2_ and CPAP reduction to room air) and post test (room air). Monitoring of transcutaneous CO_2_ was added to SpO_2_ and the newborns passing the test were left in room air.

**Results:**

Six of 23 tested babies (26%) passed the challenge at 28 days of life, 4 of 10 tested babies (40%) passed the challenge at 36 weeks. Median values of SpO_2_ were significantly higher in the neonates passing the test, respect to the failing patients. At the same time median values of TcPCO_2_ were significantly higher in the latter babies.

**Conclusion:**

TcPCO_2_ monitoring appeared to be a new useful parameter for failure prediction of weaning. The test represented a clinical guide because the newborns passing it were left in room air.

## Introduction

A reliable assessment of the premature infant’s ability to maintain adequate, independent ventilation in room air is likely to depend on an understanding of the complex interaction of respiratory system compliance, airway resistance, central inspiratory drive, chest wall stability and diaphragmatic endurance. In our previous study we showed in the babies successfully extubated significantly higher values of spontaneous expiratory minute ventilation and of respiratory rate with significantly lower transcutaneous partial pressure of CO_2_ (TcPCO_2_) values during a 2 h endotracheal tube continuous positive airways pressure (CPAP) trial, respect to the babies who failed extubation attempt [1]. On the basis of this experience, we decided to perform a new study to verify if a physiologic test, modified respect to that developed by Walsh and colleagues [2] for estimating BPD rate, could also be used as a clinical tool for weaning the premature babies from CPAP and/or oxygen therapy.

Most of the neonates with BPD have their pulmonary disease defined clinically on the basis of oxygen dependence at 36 weeks of postmestrual age (PMA). The validity of this definition is limited because the need for oxygen is determined not uniformly by individual clinician [3] and there are no standard criteria for its use. Moreover, the immaturity of respiratory control can influence oxygen use, medications and respiratory support can influence the need for oxygen, last but not least the use of oxygen at a specific time does not reflect a chronic stage [4].

To minimize the influence of different strategies for oxygen supplementation on the incidence of BPD, Walsh et coll. Proposed a physiologic test to standardize the need for oxygen at the time when BPD is diagnosed [2]. The use of this test, based on gradual oxygen weaning to room air in selected infants receiving low amounts of oxygen, made it clear that part of the reported variation in BPD incidence among centres was due to differences in clinical practice with respect to oxygen therapy [5]. BPD was originally described by Northway in a group of preterm infants suffering from respiratory distress syndrome, who develop chronic respiratory failure and radiographic pulmonary changes after prolonged mechanical ventilation and high inspiratory oxygen levels [6]. This severe form of BPD has been replaced in the last years by a milder form that presents in the smaller infants who frequently have only mild or no initial respiratory distress [7], described as “new BPD” [4].

The aim of our study was to verify if a modified physiologic test can also be used as a clinical tool for weaning the premature babies from respiratory support and/or oxygen therapy. Several innovations characterized our study: 1) the test was performed also in the babies receiving CPAP, 2) even at 28 days of life (DOL) and not only at 36 weeks PMA, 3) the newborn infants passing the test were left in room air; 4) transcutaneous gas monitoring was added to the clinical observation, SpO_2_, heart rate and respiratory rate monitoring.

## Materials and methods

### Study population

This prospective cohort study was carried out in our neonatal intensive care unit (NICU) over a 2-year period. Neonates with birth weight (BW) between 500 g and 1250 g and with gestational age (GA) ≤ 32 weeks were studied at 28 DOL and at 36 weeks PMA. Neonates receiving at that times mechanical ventilation, or CPAP and/or O_2_-therapy with fraction of inspired oxygen (FiO_2_) > 0.30, were not performing the physiologic test.

Neonates on CPAP and/or on oxygen therapy with FiO_2_ ≤ 0.30 at rest, with O_2_ saturation between 90 and 96%, underwent a timed stepwise reduction of CPAP (for the babies managed with 6 cm H_2_O first reduction to 4 cm H_2_O and then suspension of CPAP; for the babies managed with 4 cm H_2_O direct suspension of CPAP) and/or FiO_2_ to room air. Testing was done at least 1 h after feeding while the infant was quiet and in the supine position and it was performed into three steps: baseline, challenge and post test. Baseline lasted 30 min, in which the babies were observed at their initial respiratory parameters. After it, challenge based on 2% FiO_2_ reduction every 15 min till room-air was obtained. If the babies reached room-air, they underwent the post test based on a 60 min period of observation at FiO_2_ 0.21. If the baby was on nasal CPAP, challenge consisted on the suspension of CPAP. During the time test neonates were monitored continuously with a cardio-respiratory monitor (Hewlett Packard OmniCare), a pulse oxymeter (Masimo®), a transcutaneous gas monitoring (Tina TCM4, Radiometer, Copenaghen) and observed directly by an experienced neonatologist. During the test, every 60 s the following parameters were recorded: transcutaneous partial pressure of CO_2_ (TcPCO_2_), transcutaneous partial pressure of O_2_ (TcPO_2_), heart rate, respiratory rate, SpO_2_.

Test failure was defined as the occurrence of any of the following situations during any phase of the test: SpO_2_ between 80 and 89% for 5 min with TcPO_2_ < 50 mmHg, or SpO_2_ < 80% for 1 min, or apnea (cessation of breathing for > 20 s) and/or bradycardia (heart rate < 80 bpm for > 10 s). In these cases, the test was declared failed and the infant returned to the prior oxygen level immediately. The newborns passing the test were left in room air and carefully observed by a neonatologist in the following hours. The study protocol and consent forms were approved by the Ethics Committee of the Department of Pediatrics and the parents gave their informed consent.

The incidence of major morbidities (IVH > 2°; proven sepsis and patent ductus arteriosus surgically ligated) before performing the test either at 28 days and 36 weeks PMA was reported.

### Statistical analysis

Continuous variables were described by mean and SD or median and range and were compared using parametric (one-way analysis of variance-ANOVA, Unpaired t test) or non-parametric (Kruskal-Wallis, Mann-Whitney) tests, as appropriate. Categorical variables were compared using X^2^ test or a two-tailed Fisher’s exact test, as appropriate. The statistical software used was GraphPad PRISM Version 3.02. A *p* value < 0.05 was considered statistically significant.

## Results

During the study period 125 neonates with BW 500–1250 g and with GA ≤ 32 weeks were admitted to our NICU. Sixteen babies died before 28 days of life, while 61 babies were in room air and 25 were on mechanical ventilation or CPAP with FiO_2_ > 0,30, so they did not perform the test.

We performed 33 tests on 31 preterm neonates, because two neonates did the test twice: they failed it at 28 DOL, but overcame it at 36 weeks PMA. All tested infants received oxygen therapy for at least 28 days, according to definition of new BPD.

**Fig. 1:**
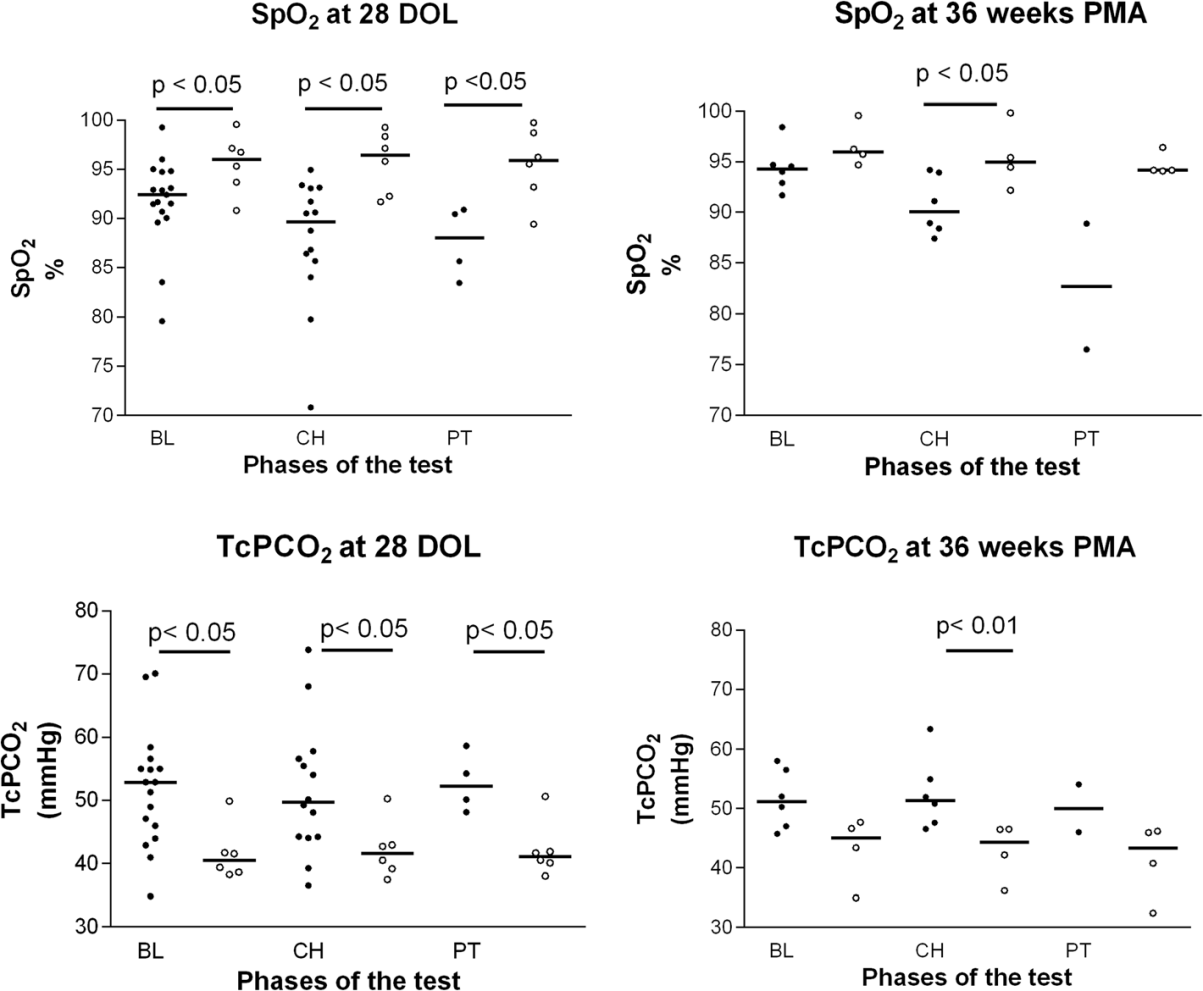
Recorded parameters at 28 days of life (DOL) and at 36 weeks post menstrual age (PMA) in the two groups of infants during the three phases of the test. BL = Baseline; CH = Challenge; PT = Post-test; TcPCO_2_ = Transcutaneous partial pressure of CO_2_; ○ = Passed group; ● = Failed group. Bar shows the median value for each group. Mann Whitney test was applied for comparisons between the groups

Twenty-three neonates were examined at 28 days of life: 6 overcame the test (Passed group), while 17 failed it (Failed group). Table 1 shows patients characteristics, major morbidities and respiratory support at 28 DOL. Before the challenge, most infants of both groups were on nasal CPAP of 4–6 cmH_2_O by nasal prongs. The Failed group infants received significantly higher median FiO_2_ levels respect to the Passed group ones (*P* = 0.03). No differences were found in terms of respiratory rate or heart rate between the two groups (data not shown). The causes of failure are shown in Table 2. Six babies passed the test (3 on CPAP of 6 cmH_2_O, 2 on CPAP of 4 cmH_2_O and 1 on O_2_-therapy) and were left into room air: four did not need any respiratory support until discharge; only two started again their ventilator support, one because of bradycardia and hypoxia associated to a not proven sepsis episode and one because of hypoxia associated to pneumonia, both 48 h after passing the test. Monitored SpO_2_ and TcPCO_2_ are reported in the Fig. 1. Median SpO_2_ values were significantly higher in the Passed group respect to the Failed group in all phases of the test (*P* < 0.05). Median TcPCO_2_ values were significantly higher in the neonates failing the test respect to the Passed group at baseline, challenge and post test (*P* < 0.05). Median [range] TcPO_2_ values were always higher in the neonates passing the test respect to those who failed it, but the only significant difference was observed during the challenge phase: 69 [61–82] mmHg and 56 [37–78] mmHg respectively (P < 0.05). Only the neonates of the Passed group increased their respiratory rate, rising from median [range] values of 52 [44–65] breaths/minute at the baseline, to 62 [46–84] breaths per minute and 66 [46–82] breaths per minute during the challenge and post test, respectively (One-way ANOVA *P* = 0.12). The respiratory rate of the Failed group remained instead unchanged during the phases of the test: median [range] values of 51 [35–73] breaths/minute at the baseline, 51 [33–86] breaths per minute during the challenge and 52 [39–66] breaths/minute during the post test. No significant respiratory rate differences between Passed and Failed groups were found during the three phases of the test.

**Table 1:** Patients characteristics, major morbidities and respiratory support at 28 DOL

	All infants (N 125)	Died infants (N 16)	Non tested infants (room air) (N 61)	Non tested infants (MV or FiO_2_ > 0.3) (N 25)	Failed Group (N 17)	Passed Group (N 6)	P_1_	P_2_
GA (wks)	28.0 ± 2.5	26.5 ± 1.5	29.8 ± 2.0	26.1 ± 1.4	26.5 ± 1.7	26.5 ± 1.8	< 0.0001	0.89
BW (g)	923 ± 234	752 ± 231	1061 ± 140	740 ± 227	856 ± 215	917 ± 187	< 0.0001	0.54
M/F	62/63	9/7	24/37	16/9	9/8	4/2	0.22	0.66
RDS	85 (68)	16 (100)	28 (46)	24 (96)	12 (70)	5 (83)	< 0.0001	1
PDA	15 (12)	2 (12)	0	7 (28)	5 (29)	1 (17)	0.0007	1
IVH > 2^°^	14 (11)	3 (19)	2 (3)	7 (28)	3 (18)	0	0.01	0.54
Sepsis	37 (30)	6 (37)	13 (21)	11 (44)	5 (29)	2 (33)	0.28	1
Survival	104 (83)	0	61 (100)	21 (84)	16 (94)	6 (100)	0.01	1
Neonates on CPAP	26 (21)		0	7 (28)	14 (82)	5 (83)	< 0.0001	1
Baseline FiO_2_	0.21 [0.21–0.55]		0.21 [0.21–0.21]	0.30 [0.21–0.55]	0.25 [0.21–0.30]	0.21 [0.21–0.23]	< 0.0001	0.03

Values are expressed as mean ± SD, median [range] or number (%)

P_1_: comparison between the groups; P_2_: comparison between the Failed and Passed groups

*GA* gestational age, *BW* birth weight, *RDS* respiratory distress syndrome, *PDA* patent ductus arteriosus surgically ligated, *IVH* intraventricular hemorrhage, *FiO*_*2*_ Fraction of inspired oxygen, *MV* mechanical ventilation, *CPAP* continuous positive airways pressure, *MV* mechanical ventilation

**Table 2:** Causes of failure of the test at 28 DOL

Cause of failure	Baseline (N 3)	Challenge (N 10)	Post-test (N 4)
Apnea	0	0	1
Bradycardia	1	0	1
SpO_2_ 80–90% with TcPO_2_ < 50 mmHg for 5’	0	4	1
SpO_2_ < 80% for 1’	2	6	1

At 36 weeks PMA 92 babies were in room air and 7 were on mechanical ventilation or CPAP with FiO_2_ > 0,30, so they did not perform the test. Ten neonates were tested: 4 overcame the test (Passed group) and 6 failed it (Failed group). By applying the physiological definition there was a reduction of BPD incidence from 15.6% (17/109) to 11.9% (13/109), and a reduction of severe BPD from 7.3% (8/109) to 6.4% (7/109). Table 3 shows patients characteristics, major morbidities and respiratory support at 36 weeks PMA. One out of 4 babies (25%) of Passed Group was on nasal CPAP of 4 cmH_2_O at the time of the test. The newborns of the Failed group needed a significantly higher median FiO_2_ level respect to the Passed group (*P* = 0.01). No differences were found in terms of respiratory rate or hearth rate between the two groups. The causes of failure of the test are shown in Table 4. Only one baby of the 4 passing the test (1 on CPAP and 3 on O_2_-therapy) was left without respiratory support until discharge, whereas 3 started it again: in one case after 3 days because of hypoxia associated with anaemia, in the other two cases after several days because of hypoxia and bradycardia associated with a proven sepsis. Figure 1 shows the values of monitored SpO_2_ and TcPCO_2_ during the three different phases of the test. Median SpO_2_ values were always higher in the neonates passing the test respect to the Failed group and the difference reached statistical significance only during the challenge phase (*p* < 0.05). Median TcPCO_2_ values were significantly higher in the Failed group as compared to the Passed group only during the challenge phase (*P* < 0.01). Median TcPO_2_ values were always higher, but without statistical significance, in the neonates passing the test respect to those who failed it. The neonates of the Failed group decreased over time their respiratory rate, from median [range] values of 64 [52–73] breaths/minute at the baseline, to 55 [42–74] breaths per minute and 44 [39–49] breaths per minute during the challenge and post test, respectively (One-way ANOVA *P* = 0.17), while the infants passing the test had their respiratory rate unchanged during the three test phases: median [range] values of 52 [40–57] breaths/minute at the baseline, 59 [49–65] breaths per minute during the challenge and 53 [40–72] breaths per minute during the post test. No significant respiratory rate differences between Passed and Failed groups were found during the three phases of the test.

**Table 3:** Patients characteristics, major morbidities and respiratory support at 36 weeks PMA

	Non tested infants (room air) (N 92)	Non tested infants (MV or FiO_2_ > 0.3) (N 7)	Failed Group (N 6)	Passed Group (N 4)	P_1_	P_2_
GA (wks)	28.7 ± 2.4	25.9 ± 2.0	25.5 ± 1.6	26.0 ± 0.8	< 0.0001	0.36
BW (g)	995 ± 191	667 ± 274	673 ± 141	752 ± 245	< 0.0001	0.53
M/F	41/51	5/2	4/2	3/1	0.30	1
RDS	53 (58)	6 (86)	6 (100)	4 (100)	0.03	0.40
PDA	6 (6)	3 (43)	2 (33)	2 (50)	0.0007	1
IVH > 2^°^	8 (9)	2 (28)	0 (0)	1 (25)	0.20	0.40
Sepsis	29 (31)	5 (71)	4 (67)	2 (50)	0.06	0.50
Survival	91 (99)	4 (57)	6 (100)	3 (75)	< 0.0001	0.40
Neonates on CPAP	0	1 (14)	0	1 (25)	0.0002	1
Baseline FiO_2_	0.21 [0.21–0.21]	0.30 [0.21–0.50]	0.25 [0.23–0.27]	0.23 [0.21–0.23]	< 0.0001	0.01

Values are expressed as mean ± SD, median [range] or number (%)

P_1_: comparison between the groups; P_2_: comparison between the Failed and Passed groups

*GA* gestational age, *BW* birth weight, *RDS* respiratory distress syndrome, *PDA* patent ductus arteriosus surgically ligated, *IVH* intraventricular hemorrhage, *FiO*_*2*_ Fraction of inspired oxygen, *MV* mechanical ventilation

**Table 4:** Causes of failure of the test at 36 weeks PMA

Adverse event	Baseline (N 0)	Challenge (N 4)	Post-test (N 2)
Apnea	0	0	0
Bradycardia	0	0	0
SpO_2_ 80–90% with TcPO_2_ < 50 mmHg for > 5’	0	2	1
SpO_2_ < 80% for 1′	0	2	1

## Discussion

The physiologic test proposed by Walsh is a structured, short period of oxygen saturation monitoring coupled with gradual oxygen weaning to room air in selected infants receiving low amounts of oxygen [2, 5]. In our study we performed this test with four innovations. First of all, we performed the test also at 28 DOL. Older studies defined BPD as treatment with supplemental oxygen for longer than 28 to 30 days [8, 9], whereas more recent studies have defined BPD as treatment with supplemental oxygen at 36 weeks PMA [10, 11]. Predictive values of oxygen for ≥28 days, oxygen at 36 weeks PMA, and the severity-based NIH consensus definition of BPD [12] were recently evaluated in a large cohort of infants from NICHD at 18 to 22 months corrected age [13]. The 28 days of oxygen criteria is more sensitive in detecting post discharge respiratory complications, but has poor specificity. The oxygen dependency at 36 weeks PMA is more specific and more sensitive in predicting mental and psychomotor developmental impairment [4, 13]. It has to be taken into account that most of our patients evaluated at 28 DOL suffered from RDS at birth and that all needed a respiratory support and/or O_2_-therapy continuously during the first month of life. Oxygen, which is vital to survival, can be highly damaging to neonatal tissue which is known to be poorly equipped to neutralize toxic derivatives. Recently a marker of oxidative stress in preterm newborns was found [14]. Preterm infants are often exposed to high oxygen concentrations, infections or inflammation; they have reduced antioxidant defense and high free iron levels which enhance toxic radical generation [15]. Using lung protective strategies or by performing tests like ours, coupled with gradual oxygen weaning to room air in selected infants receiving low amounts of oxygen, could be very useful to prevent oxidative induced lung injury in preterm infants. Second, the test was performed also in neonates receiving CPAP when their FiO_2_ was ≤30%. It was very interesting that 5 of the six neonates that overcame the test at 28 DOL were on CPAP, and only 2 started again their respiratory support in the following days, while at 36 weeks PMA of the 4 neonates passing the test only one was on CPAP and he started again his respiratory support in the following days. The possibility of suspending both O_2_-therapy and nasal-CPAP, represents an undeniable gain not only for newborn’s welfare, but also for enhancing the sound of material and human resources. Moreover weaning from CPAP at 36 weeks PMA could impact the rate of severe BPD (“new BPD”). In our centre the proportion of infants requiring mechanical ventilation is very low, so by applying the physiological definition there was a reduction of severe BPD from 7.3% (8/109) to 6.4% (7/109).

Third, we introduced the transcutaneous gas monitoring. In particular, TcPCO_2_ could be a good criterion for failure’s prediction, being always significantly higher in the Failed group. In infants with BPD, hypercapnia reflects the presence of a more severe lung disease and may be the result of a combination of ventilation/perfusion (V/Q) imbalance, hypoventilation, and diaphragmatic fatigue [16–18]. There is also evidence that infants with BPD have an impaired respiratory response to pCO_2_ [19] as showed by respiratory rate behaviour. In addition the presence of hypercapnia suggests reduced ventilator reserve, with an increased risk of ventilator failure in the presence of added mechanical or metabolic stress [17].

Last but not least, we intended to use the test also to guide clinical decision about an individual’s patient care, so we modified the length of each phase of the test. The baseline period, which lasted 15 min in Walsh’s study was lengthened to 30 min. During the challenge phase, each step of reduction lasted 15 min instead of the 5 decided by Walsh, to better evaluate the effects of each reduction on baby’s condition and saturation. Moreover we performed a longer observation in room air: 60 min in our study vs 30 min in Walsh’s experience. Someone may argue that in this way the test required more than 2 h to be performed, loosing its feasibility. Nevertheless, the median length of test in failed group was 53 min [9–104] and 50 min [40–135], respectively at 28 DOL and at 36 weeks PMA, demonstrating the need of a longer duration of the physiologic challenge. It is important to note that the neonates passing the test but requiring respiratory support and/or O_2_-therapy in the following days were all affected by non-respiratory (mainly infective) diseases. This underlines the utility of our test as a clinical tool for weaning preterm newborns from their respiratory support.

In our study, the Passed and Failed groups were similar with respect to gestational age, birth weight and major morbidities. The infants who failed the test had lower median SpO_2_ values compared to the babies who have passed the test, while receiving a significantly greater FiO_2_. These data underline the alteration of V/Q relationship, one of the characteristics of BPD; in fact non-uniform damage to the airways and distal lungs results in variable time constants for different areas of the lungs, the inspired gas might be distributed to poorly perfused lungs, and worsening ventilation-perfusion matching [20]. Of the other monitored parameters, median TcPCO_2_ values were always higher in the Failed group during the three phases of the test. It is important to note that at 28 DOL 19/23 studied infants (83%) were on nasal CPAP. During the challenge and post test phases, it is conceivable that infants deprived of any respiratory support, should provide themselves to maintain a functional residual capacity, which can be implemented by increasing the respiratory rate. While the neonates of Passed group increased their respiratory rate, infants of Failed group maintained a respiratory rate essentially unchanged during the three phases of the test, despite the significantly higher values of TcPCO_2_. It is possible that the babies of the Failed group had a tendency to breathe with less efficiency respect to the babies of Passed group, for a combined effect of more severe lung injury (as shown by their higher FiO_2_ requirement at baseline), immaturity of central inspiratory drive (as shown by a lower respiratory rate during the test) and impaired respiratory muscle strength.

Kovesi et al. showed that pCO_2_ values are related to an increased incidence of adverse events, defined as death, need for respiratory assistance and/or tracheotomy and pulmonary hypertension [21]. As demonstrated by Kaempf et al. in a multicenter study, pCO_2_ and SpO_2_ values appears to be reasonable good markers of lung injury [22]: the authors studied 220 babies at 36 weeks PMA, subjecting them to a challenge of 1 h to FiO_2_ 0.21. Median pCO_2_ values were significantly higher in infants with BPD compared to controls: 54 mmHg vs 45 mmHg, respectively, consistent with the results of our study.

## Conclusion

The test has proven to be safe and feasible, in fact neonates were monitored by a clinician in each step of the test, while direct observation is only intermittent in routine clinical practice during FiO_2_ weaning. We want also to underline the utility of a follow up of the neonates passing the test in order to validate it for clinical management. In contrast to the method of Walsh, in our study infants were left in room air if the challenge was passed with the intention of using the test as a clinical tool for weaning. We have shown that some infants did not need the respiratory support they were being given at that time. In our experience half of the infants who passed the test, later required respiratory support, although this can certainly not be attributed to respiratory failure.

We hope that infants kept on oxygen and/or respiratory support could be removed sooner by using our test.
